# Toll-like receptor polymorphisms and cerebral malaria: *TLR2 *Δ22 polymorphism is associated with protection from cerebral malaria in a case control study

**DOI:** 10.1186/1475-2875-11-47

**Published:** 2012-02-15

**Authors:** Jennifer A Greene, Nadia Sam-Agudu, Chandy C John, Robert O Opoka, Peter A Zimmerman, James W Kazura

**Affiliations:** 1 Case Western Reserve University, Wolstein Research Building, 2103 Cornell Rd, Cleveland, OH 44106, USA; 2 University of Minnesota, Minneapolis, MN, USA; 3 Makerere University, Kampala, Uganda

**Keywords:** Malaria, Toll-like receptor, *Plasmodium falciparum*, Polymorphism, GPI

## Abstract

**Background:**

In malaria endemic areas, host genetics influence whether a *Plasmodium falciparum*-infected child develops uncomplicated or severe malaria. TLR2 has been identified as a receptor for *P. falciparum*-derived glycosylphosphatidylinositol (GPI), and polymorphisms within the TLR2 gene may affect disease pathogenesis. There are two common polymorphisms in the 5' un-translated region (UTR) of TLR2, a 22 base pair deletion in the first unstranslated exon (Δ22), and a GT dinucleotide repeat in the second intron (GTn).

**Methods:**

These polymorphisms were examined in a Ugandan case control study on children with either cerebral malaria or uncomplicated malaria. Serum cytokine levels were analysed by ELISA, according to genotype and disease status. In vitro TLR2 expression was measured according to genotype.

**Results:**

Both Δ22 and GTn polymorphisms were highly frequent, but only Δ22 heterozygosity was associated with protection from cerebral malaria (OR 0.34, 95% confidence intervals 0.16, 0.73). In vitro, heterozygosity for Δ22 was associated with reduced pam3cys inducible TLR2 expression in human monocyte derived macrophages. In uncomplicated malaria patients, Δ22 homozygosity was associated with elevated serum IL-6 (*p *= 0.04), and long GT repeat alleles were associated with elevated TNF (*p *= 0.007).

**Conclusion:**

Reduced inducible TLR2 expression may lead to attenuated pro-inflammatory responses, a potential mechanism of protection from cerebral malaria present in individuals heterozygous for the TLR2 Δ22 polymorphism.

## Background

In some holoendemic countries, individuals are bitten by malaria-infected mosquitoes 60-300 times a year [1,2]. The most common form of malaria in endemic countries is uncomplicated malaria, characterized by fever, chills, sweats, headaches and nausea. Cerebral malaria is a severe neurologic complication of *Plasmodium falciparum *infection that occurs much less commonly than uncomplicated malaria. Genetic factors influence infection outcome, and polymorphisms in genes encoding proteins involved in disease pathogenesis are strong candidates for disease association studies. As a receptor for parasite derived GPI, TLR2 is likely involved in disease pathogenesis [3], however, a previous study examining single nucleotide polymorphisms (SNPs) within TLR2 could not detect an association with severe disease because these SNPs were absent in the populations studied [4]. Reportedly, TLR2 SNPs are absent in some African countries as well, but insertion deletion polymorphisms within the 5' un-translated region of TLR2 are very common [5,6]. A 22 bp insertion/deletion polymorphism (*TLR2 *Δ22) in the first un-translated exon was highly polymorphic in Kenyans and in a Japanese population but was not associated with a disease phenotype [5,7]. A GT dinucleotide repeat that varies by approximately 12 to 30 repeats (GT_n_) is present within the second intron, approximately 100 bp upstream of the translational start site. Repeats of varying length have been associated with susceptibility to tuberculosis, reversal reactions in leprosy and colorectal cancer [8-10]. These polymorphisms are associated with altered in vitro phenotypes. The TLR2 Δ22 polymorphism was associated with reduced constitutive luciferase reporter activity compared to a construct containing the insertion allele [7]. Shorter GT_n _repeats are associated with reduced TLR2 reporter activity and TLR2 surface expression in vitro [8]. Altered TLR2 expression in individuals with different TLR2 polymorphisms may lead to differential pro-inflammatory responses based on genotype. TLR2 expression on human monocytes ex vivo is highly variable [11]. Reports on pam3cys and LPS inducible TLR2 expression on human monocytes vary, likely due to donor specific differences as monocytes differentiate into macrophages [11,12]. *Plasmodium falciparum *derived GPI activate TLR2 to induce TNF, IL-12, IL-6 and nitric oxide in murine bone marrow derived macrophages [3]. Elevated levels of IFN-γ, TNF, IL-12, IL-6, IL-1β, and IL-10 are initially protective, although excessive serum levels of these cytokines are associated with cerebral malaria pathology [13-15]. Similarly, IFN-γ and TNF are necessary for disease in the murine model of cerebral malaria [16-19]. In the children examined in this study, cerebral malaria patients had elevated serum levels of IFN-γ, IL-1β, and IL-10 compared to uncomplicated malaria patients [20]. Although neither IL-6 nor TNF serum levels were correlated with severe disease in these children [20] these cytokines are often associated with severe disease in other epidemiologic studies [13].

In the current study, the role of TLR2 insertion/deletion polymorphisms, Δ22 and GT_n_, in the pathogenesis of cerebral malaria was evaluated. Genomic DNA and serum were collected from Ugandan children participating in a case control study examining children with cerebral malaria and uncomplicated malaria. This data indicates heterozygosity for Δ22 polymorphism was protective (odds ratio 0.34, 95% CI 0.16-0.73), but there was no disease association with the GT_n _polymorphism. In malaria naïve donors, Δ22 heterozygosity was associated with reduced pam3cys induced TLR2 stimulation in human monocyte derived macrophages. In Ugandan children involved in the case control study, none of these polymorphisms was associated with serum cytokines in the cerebral malaria group. In the uncomplicated malaria group, homozygosity for the Δ22 insertion allele was associated with elevated IL-6 (*p *= 0.04), and at least one long GT repeat allele was associated with elevated serum TNF levels (*p *= 0.007). This data suggests that the TLR2 insertion deletion polymorphisms may be involved in cerebral malaria pathogenesis through alteration of induced TLR2 expression and down-regulation of specific pro-inflammatory cytokines.

## Methods

Children aged three-12 years were recruited for a study assessing complications of cerebral malaria, which was conducted at Mulago Hospital in Kampala, Uganda [20]. Eighty-five children with cerebral malaria (CM) and 76 children with uncomplicated malaria (UM) were recruited. Children with CM were included in the study if they were admitted to Mulago Hospital, and met the WHO criteria for CM: coma (Blantyre coma scale ≤2, Glasgow coma scale ≤8), *P. falciparum*-smear positive, and no other signs of encephalopathy. Lumbar puncture was performed to rule out meningitis or encephalitis. Children with UM were enrolled at the acute-care clinic or a malaria outpatient clinic that is sponsored by the University of California San Francisco (UCSF). Children were enrolled as UM patients if they exhibited the signs and symptoms of malaria (fever, chills, vomiting, headache), were *P. falciparum*-smear positive, had no malaria-related complications (e.g. seizures, coma, severe anaemia, or respiratory distress), no other sign of acute illness, and were not hospitalized for their malarial illness. Details of study enrolment have been reported previously [20]. Genetic testing was requested from study participants upon follow-up testing at two years after enrolment. If consent was obtained from the study participant parent or guardian, testing for TLR polymorphisms was performed on the filter paper samples collected at initial enrolment.

Exclusion criteria for children in all three groups was (1) a history of meningitis or encephalitis, or any other brain disorder (including CM); (2) a history of developmental delay; (3) prior admission to the hospital for malnutrition; (4) a history of chronic illness. Ethical approval for these studies was granted by the institutional review boards for human studies at Makerere University Faculty of Medicine, University Hospitals of Cleveland, Case Western Reserve University, Indiana Wesleyan University, and the University of Minnesota. Upon admission, study participants' blood was collected onto Whatman filter paper (Whatman Corporation, Florham Park, NJ). Genomic DNA was extracted from dried blood spots using the QIAamp 96 spin blood kit (QIAGEN, Valencia, CA). Presence of *P. falciparum *was determined by light microscopy of thin and thick blood smear with two independent readings. A third independent reading was performed if necessary to resolve any discrepancies between the initial two readings.

### Polymerase chain reaction (PCR)

Genomic DNA was extracted from blood using the QIAamp 96 spin blood kit (QIAGEN, Valencia, CA). PCR was performed using a master mix consisting of 1× PCR buffer, 125μM dNTPs, 2.5 mM MgCl_2_, 125nM primers, and 0.8 units *Taq *polymerase in a reaction volume of 25μl. PCR primers and amplification conditions have been described previously [5]. *TLR2 *Δ22 genotypes were assigned based on size discrimination of PCR products on a 4% agarose gel. *TLR2 *GT_n _genotypes were assigned based on size discrimination of PCR products run on a 6% polyacrylamide gel as described elsewhere [21].

### Cloning and sequencing

PCR amplification products from local donors were purified using the QIAquick PCR purification kit (QIAGEN, Valencia, CA). Purified PCR products were sent to MWG Biotech, High Point, NC for sequencing. Sequences were analysed using the Sequencher software (Gene Codes Corporation, Ann Arbor, MI).

### Cytokine testing

Serum levels of IFNγ, TNF, IL-1β, IL-10, and IL6 in children with uncomplicated malaria and cerebral malaria were compared according to TLR2 genotype. Serum samples for cytokine measurement were obtained at the time of admission (CM) or outpatient enrollment (UM). Cytokine testing was done using cytometric bead array (CBA) technology using the Bioplex-Luminex system (Austin TX), or ELISA as previously described [6,20].

### Statistical analysis

Allele and genotype frequencies were calculated for the different polymorphisms, and the Hardy-Weinberg exact test (estimation of p-values by the Markov chain method) was performed for each population using Arlequin version 3.01[22]. Haplotypes were statistically inferred using the Expectation - Maximization method in Arlequin. Chi squared test was used to determine allelic, genotypic, and haplotypic associations using SPSS for Windows (version 13.0). Genotypic tests were performed for additive, dominant, recessive, and heterozygote advantage models. Odds ratios were performed for the different genotype models using an online calculator[23]. All cytokines were log transformed to create a normalized distribution, and comparisons for the different genotype models were made using the *T*-test using SPSS for windows (version 13.0). Because of errors due to multiple comparisons, Bonferroni correction assuming two individuals tests was applied. At a false positive rate of 5%, the adjusted significance threshold becomes *p *< .025.

### TLR2 expression by flow cytometry

Venous blood was collected from 18 malaria naïve North American volunteers. Peripheral blood mononuclear cells were isolated by Ficoll-Paque separation (GE Healthcare, Piscataway, NJ) and stained with TLR2-FITC (clone TL2.1) and CD14-APC (clone 61D3, eBiosciences, San Diego, CA). Cells were read on an LSRII flow cytometer (BD Biosciences, Franklin Lakes, NJ). Monocytes were distinguished by scatter characteristics and CD14 positivity. Median fluorescence intensity of isotype controls were subtracted from each sample to determine relative TLR2 fluorescence. Ethical approval for these studies was granted by the institutional review board for University Hospitals of Cleveland, Case Western Reserve University.

### Cell culture

Peripheral blood mononuclear cells (PBMC) were cultured in RPMI 10% human serum AB, 2 mM L-glut, 20 mM HEPES, and pen/strep. PBMC were plated out at 1 × 10^6 ^cells/ml and stimulated with Pam3Cys (200 ng/ml) for 24 h. Human monocytes were isolated by immunomagnetic purification using the Miltenyi monocyte isolation kit II (miltneyi) to obtain unlabelled monocytes which were 80-95% pure. Purified monocytes were cultured for six days in RPMI supplemented with 2 mM L-glut, 20 mM HEPES, pen/strep, and 10% autologous serum to allow differentiation into monocyte derived macrophages. Cells were washed three times with PBS, and adherent cells were removed with cell dissociation buffer (vendor) for flow cytometry.

### Parasite culture

*Plasmodium falciparum *strain 3D7 parasites were cultured and the parasite life cycle was synchronized as described [24]. Trophozoites and schizonts were obtained by magnetic purification. Late stage parasite cultures were washed three times with Macs buffer (PBS, pH 7.2, 0.5% FBS, and 2 mM EDTA), and resuspended at a concentration of 2 × 10^9 ^cells/ml. Parasite cultures were passed through Miltenyi LD columns (Miltenyi), allowing uninfected RBCs and rings to pass through the column. Miltenyi columns were washed two times 1 ml Macs buffer, and late stage parasites were eluted with 3 ml Macs buffer. Trophs/schizonts were washed three times and counted. Schizonts were lysed by sonication (40 V, 10 mins). Following sonication, schizonts were brought up to the appropriate concentration in RPMI. Schizonts or intact trophozoites were added to macrophage cultures at a ratio of 10:1.

### RT-qPCR

Approximately 1 × 10^6 ^monocytes were isolated for qPCR. Total RNA was extracted using the PureLink RNA Mini Kit (Invitrogen). 100 ng of total RNA was reverse transcribed into cDNA using commercial reagents (Invitrogen) and non-specific Oligo-DT primers. Quantitative PCR was carried out using the following gene specific primers; TLR2 - forward 5'-ATTGTGCCCATTGCTCTTTC-3', reverse, 5'-CTTCCTTGGAGAGGCTGATG-3'; GAPDH - forward 5'-AAGATCATCAGCAATGCCTCCTGC-3', reverse 5'-ATGGACTGTGGTCATGAGTCCTTC-3'. Samples were analysed on a 7300 Real Time PCR System (Applied Biosystems), and normalized using the comparative threshold method [25,26]. HEK cell mRNA was used as a negative control for TLR2 expression.

## Results

### *TLR2 *Δ22 and GT_n _genetic association with cerebral malaria

**Figure 1:**
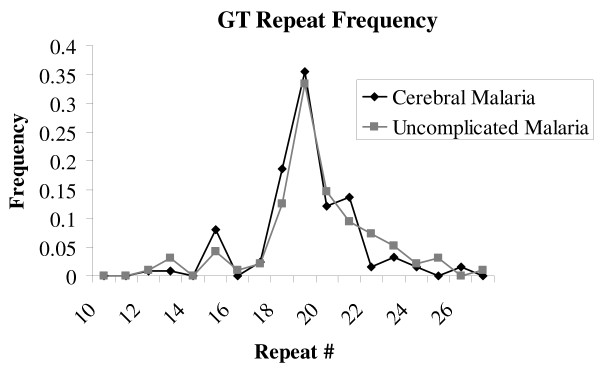
**GT repeat allele frequencies in Ugandan children**.

A total of 65 children with cerebral malaria and 52 children with uncomplicated malaria were genotyped for *TLR2 *insertion-deletion polymorphisms. The Δ22 polymorphism was highly polymorphic in this study, but there was no significant difference in allele frequency between the two groups (0.27 in the cerebral malaria group vs 0.35 in the uncomplicated malaria group, *p *= 0.20). The GT_n _polymorphism was highly polymorphic but similarly distributed between the two groups, as demonstrated in Figure 1.

*TLR2 *Δ22 and GT_n _genotype frequencies are displayed in Table 1. The heterozygote advantage genotype model best fits the Δ22 polymorphism, as heterozygosity was significantly more common in the uncomplicated malaria group compared to the cerebral malaria group (WD vs DD/WW, *p *= .005 OR 0.34, 95%CI 0.16-0.73), and this was below the adjusted significance threshold of *p *< 0.025. Additionally, in the uncomplicated malaria group there was a slight deviation from Hardy Weinberg Equilibrium (*p *= 0.065), indicating an excess of heterozygotes in this group. Consistent with how the GT_n _repeats have been analysed previously [8,27], the alleles were classified as short (S < 16), medium (M, 16-23), and long (L > 23) repeats. All individuals possessed at least one medium length repeat allele. Only SM, MM, and ML genotypes for the GT_n _repeat polymorphism were present in the groups studied here, and these genotypes were similarly distributed between cerebral malaria patients and controls with uncomplicated malaria (Table 1). *TLR2 *haplotype frequencies are presented in Table 2. All six possible *TLR2 *haplotypes were present, and similarly distributed in each group. There was no linkage disequilibrium between these two polymorphisms, as defined by both a Lewontin's D' and r^2 ^value >0.8 (UM D' = 0.553, r^2 ^= 0.083; CM D' = 0.490, r^2 ^= 0.094).

**Table 1:** *TLR2 *Δ22, and GT_n _genotypes

Polymorphism	Genotype	Cerebral malaria n = 65	Uncomplicated malaria n = 52	*p *value	OR	95% CI
Δ22	W/W	36(56.3)	18(35.3)	.025^I^	0.42	0.20-0.98
	W/D	21(32.8)	30(58.8)^II^	.005^III^	0.34	0.16-0.73
	D/D	7(10.9)	3(5.9)			
GT_n_	SM	12(19.4)	8(16.7)			
	MM	46(74.2)	34(70.8)			
	ML	4(6.4)	6(12.5)			

OR and 95% CI presented only for p values where *p *< 0.05

^I ^p value for dominant genotype test, WD/DD vs WW

^II^*p *= 0.065 for Hardy Weinberg Test of equilibrium, excess of heterozygotes

^III ^*p *value for heterozygote advantage genotype test, WD vs DD/WW

**Table 2:** *TLR2 *haplotypes

Haplotype	Cerebral malaria	Uncomplicated malaria
D S	0.078	0.083
D M	0.185	0.265
D L	0.000	0.006
W S	0.021	0.000
W M	0.684	0.589
W L	0.033	0.057

D, deletion allele, W, wild type

S < 16 repeats, M 16-23 repeats, L > 23 repeats

### Constitutive TLR2 expression on human monocytes and monocyte derived macrophages

**Figure 2:**
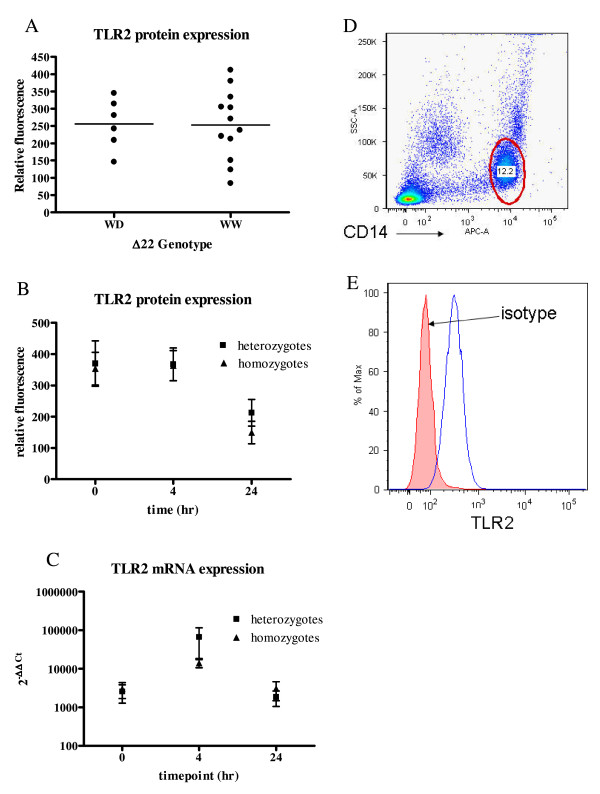
**Constitutive TLR2 expression on primary monocytes**. TLR2 expression on CD14+ cells immediately ex vivo or during the first 24 h in culture is shown here. Figure A is TLR2 expression on CD14+ cells ex vivo according to Δ22 genotype. Relative fluorescence is median fitc-TLR2 fluorescence minus median fitc-isotype fluorescence for each individual. Horizontal bars indicate median for each group, and each dot represents one donor. Representative histograms and the gate used to identify monocytes is represented in D and E. Figure B shows TLR2 protein expression on immunopurified monocytes during the first 24 h in culture according to Δ22 genotype. Error bars indicate mean +/− SEM. There are four heterozygotes and four homozygotes are in each group. Figure C shows the corresponding TLR2 mRNA expression in the first 24 h of culture according to *TLR2 *Δ22 genotype. Relative mRNA expression was quantified according to the comparative Ct method, using GAPDH as the housekeeping gene, and HEK cell mRNA as a negative control. In graph C the results at 0 h for one heterozygous donor was removed, this person was an outlier, with a 2^-ΔΔCt ^value of 3.3 × 10^8^.

Malaria naïve North American volunteers were recruited and genotyped for TLR2 Δ22 and GT_n _polymorphisms. TLR2 expression was highly variable on monocytes from local donors ex vivo, and was not associated with Δ22 genotype in this study (Figure 2). Because of the few donors with small or large GT repeat polymorphisms in this study, the effect of this polymorphism on TLR2 expression on human monocytes ex vivo could not be determined. Reportedly, TLR2 expression on monocytes varies in the first 24 h of culture, as the cells become adherent and begin differentiating into macrophages [11,12,28]. To test whether differentiation dependent TLR2 expression was based on TLR2 Δ22 genotypes, TLR2 mRNA and protein expression were determined on monocytes at 0, 4, and 24 h in culture. Consistent with previous reports, TLR2 expression varied during the first 24 h in culture, but expression levels were not associated with genotype (Figure 2).

### Inducible TLR2 expression

**Figure 3:**
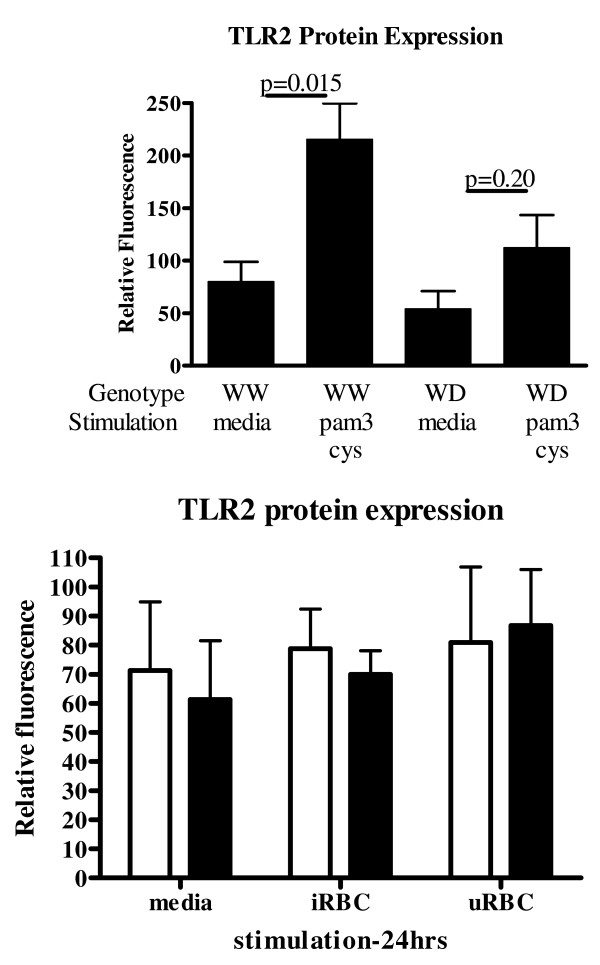
**Inducible TLR2 expression on macrophages according to TLR2 Δ22 genotype**. Human monocyte derived macrophages were stimulated with pam3cys (200 ng/ml), schizont infected RBCs (3 × 10^6^/well), or uninfected RBCs (3 × 10^6^/well) for 24 h. TLR2 protein expression is expressed as relative fluorescence. Top panel n = 5 homozygotes, n = 5 heterozygotes, bottom panel, n = 4 homozygotes, n = 5 heterozygotes. Error bars represent standard error of the mean.

Because of the variability in TLR2 expression during the first 24 h of monocyte culture, constitutive and inducible TLR2 expression on human monocyte derived macrophages was examined. Human monocytes were isolated by negative selection and cultured for six days to allow differentiation into macrophages. Although constitutive TLR2 expression was not associated with Δ22 genotype (Figure 3), a 24-h Pam3Cys stimulation significantly up-regulated TLR2 expression on macrophages from homozygous donors (*p *= 0.023), but not on macrophages from heterozygous donors (*p *= 0.20). *P. falciparum *strain 3D7 parasites were synchronously cultured to trophozoite stage and added to human monocytes derived macrophages and incubated for 24 h. Neither intact, infected RBCs or uninfected RBCs up-regulated TLR2 expression on human monocyte derived macrophages (Figure 3). Lysed infected RBCs were also unable to up-regulate TLR2 expression on human monocyte derived macrophages (data not shown).

### Serum cytokine levels in children with different TLR2 polymorphisms

**Figure 4:**
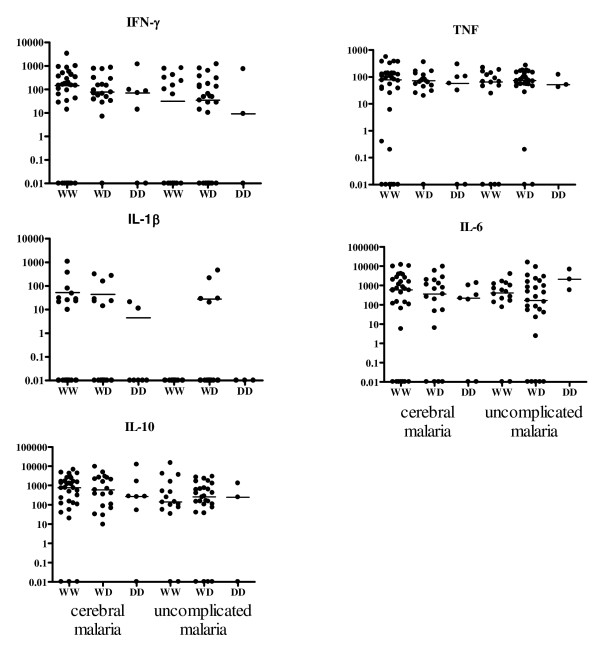
**Cytokine levels according to Δ22 genotype in children with cerebral malaria or uncomplicated malaria**. Cytokine levels are present as pg/ml, horizontal bars indicated median. 0.01 values are actually below the threshold of detection, and were considered 0 for statistical purposes.

**Figure 5:**
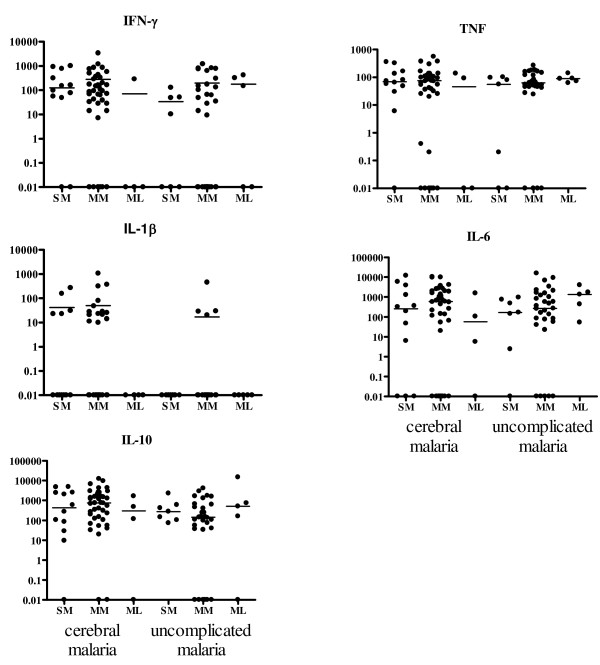
**Cytokine levels according to TLR2 GT repeat genotype in children with cerebral malaria or uncomplicated malaria**. Cytokine levels are present as pg/ml, horizontal bars indicated median. 0.01 values are actually below the threshold of detection, and were considered 0 statistically.

Serum TNF, IFN-γ, IL-1β, IL-6, and IL-10 did not vary based on either TLR2 Δ22 or GT_n _genotype in children with cerebral malaria (Figures 4 and 5 and Additional file 1: Table S1). In the uncomplicated malaria group, children with at least one long GT_n _repeat allele produced the most TNF (*p *= 0.007, Figure 5 and Additional file 1: Table S1), and children homozygous for Δ22 produced the most IL-6 compared to children with other genotypes (*p *= 0.041, Figure 4 and Additional file 1: Table S1). Only five uncomplicated malaria patients had detectable serum IL-1β levels, and they were all heterozygous for Δ22 and had medium length GT repeat alleles (Figures 4, 5, and Additional file 1: Table S1).

## Discussion

This is the first study to show an association between severe malaria and a *TLR2 *polymorphism. TLR2 Δ22 heterozygosity was significantly more common in the uncomplicated malaria group (*p *= 0.005), indicating that the odds of cerebral malaria are 66% (odds ratio 0.34, 95% CI 0.16-0.73) lower in heterozygotes compared to either homozygote. Due to the low frequency of Δ22 homozygotes, and the small sample sizes, the dominant genotype model (*p *= 0.025, OR 0.20-0.90) is also a possibility, and future studies with larger samples sizes need to be conducted to more precisely define the role of this polymorphism in susceptibility to cerebral malaria. Future studies should investigate whether this polymorphism is protective against other forms of severe malaria, such as severe malarial anaemia. Individual co-infections may have influenced the frequencies of TLR2 genotypes observed here. The Δ22 polymorphism was associated with enhanced susceptibility to acute adenolymphangitis events in lymphatic filariasis-infected Papua New Guineans (Greene et al., unpublished results). Lymphatic filariasis is one of many parasitic diseases transmitted in Uganda [29] and the relatively low frequency of Δ22 homozygotes in the uncomplicated malaria group may reflect these complex selective pressures in this region.

Within the *TLR2 *gene, Δ22 is located in the first untranslated exon of *TLR2*, approximately 60 bp downstream of an NF-kB site critical for chromatin remodelling and transcription factor binding [28]. The GT_n _repeat is located approximately 100 bp upstream of the transcriptional start site, potentially affecting inclusion of the single coding exon in the transcript. Because of their location within the 5' UTR and proximity to critical cis-elements within the TLR2 gene, it is possible that Δ22 and GT_n _may affect TLR2 expression. Although short and long GT repeats were too uncommon in local donors to detect a difference in TLR2 expression, there was reduced TLR2 expression in response to pam3cys in human monocyte derived macrophages. There was a similar, although insignificant hypo-responsiveness in human monocytes from Δ22 heterozygotes (data not shown). The focus was on macrophages in this study to avoid donor specific effects on monocyte differentiation and subsequent TLR2 expression. There was no significant TLR2 up-regulation in response to either intact parasitized RBCs or schizont lysate. This does not rule out the possibility that *P. falciparum *may directly up-regulate TLR2, but it was not detected in this study, possibly because there was insufficient GPI activating TLR2 in either of the parasite preparations to up-regulate TLR2 expression in a manner similar to pam3cys induction. Future studies should examine whether purified parasite derived GPI up-regulate TLR2. None of the North American donors homozygous for Δ22, and therefore the effect of this genotype on either constitutive or inducible TLR2 expression could not be determined.

The reduced pam3cys inducible TLR2 expression on macrophages from Δ22 heterozygous donors suggests that this polymorphism may attenuate TLR2 induced signalling cascades and downstream cytokine production. To test this, serum cytokine responses were examined in the Ugandan children involved in the case control study according toΔ22 and GT_n _genotypes. There was no significant association between the TLR2 polymorphisms examined here and serum cytokines in the cerebral malaria group. In the uncomplicated malaria group Δ22 homozygosity was associated with elevated IL-6 production (*p *= 0.04), and at least one long GT_n _allele was associated with elevated serum TNF (*p *= 0.007). In these children, neither IL-6 nor TNF were correlated with severe disease [20], though the relatively small sample size may have precluded detection of all but large associations. The same limitation regarding sample size applies to the detection of elevated levels of cytokines in children with Δ22 homozygosity and cerebral malaria. This study should be repeated in an area where elevated IL-6 and TNF correlate with severe disease. It is possible that cytokine production in individuals with specific TLR2 genotypes may influence development of uncomplicated malaria, and future studies of longitudinal cohorts examining the intensity and frequency of uncomplicated malaria episodes in children with different Δ22 and GT_n _genotypes may elucidate the relationship between TLR2 genotype and infection outcome. Examining serum cytokines is non-specific, and many cells contribute to serum cytokine levels. Future studies should examine PBMCs from donors with different TLR2 genotypes and measure cytokine responses upon stimulation with intact and lysed infected RBCs. One potentially confounding variable in the current case control study would be the presence of a MAL SNP Ser180Leu. This molecule is a downstream adaptor in the TLR2 signalling pathway, and this polymorphism is associated with protection from malaria and reduced signalling in vitro [30]. The Ugandan samples were genotyped for the MAL polymorphism as described previously [5]. The SNP was present at 1.5% allele frequency in the cerebral malaria patients, and 1% allele frequency in the uncomplicated malaria group. Because of the low frequency of this SNP, it is unlikely that it had a significant effect on the serum cytokine levels observed here.

## Conclusions

Collectively, this data suggest that heterozygosity for a 22 bp deletion within the first un-translated exon of TLR2 is associated with protection from cerebral malaria, and that this protection may be due to a dampening of the pro-inflammatory response. Pam3Cys inducible TLR2 expression was significantly reduced in macrophages from heterozygous donors. Reduced surface expression may attenuate TLR2 mediated responses. Although there were no significant differences in serum cytokines according to TLR2 genotypes in children with cerebral malaria, future studies examining responses to pam3cys and parasitized RBCs in donors with different TLR2 genotypes will help identify cell responsible for cytokine production.

## Abbreviations

RBC: red blood cell; TLR: Toll-Like Receptor; GPI: glycosylphosphatidylinositol; CM: cerebral malaria; UM: uncomplicated malaria; UTR: unstranslated region; OR: odds ratio; SNP: single nucleotide polymorphism.

## Competing interests

The authors declare that they have no competing interests.

## Authors' contributions

JG performed genotyping, in vitro work, statistical analysis, and drafted manuscript. JXK and CCJ conceived and designed the study. PAZ was involved in data analysis. NSA conducted ELISAs. ROO was involved in study sample collection. All authors read and approved the final manuscript.

## Supplementary Material

Additional file 1 **Table S1**. Cytokine association tests according to *TLR2 *genotype in Uganda children with cerebral malaria or uncomplicated malaria. Click here for file
