# Supplementary material for: Toll-like receptor polymorphisms and cerebral malaria: TLR2 Δ22 polymorphism is associated with protection from cerebral malaria in a case control study
Source: Malar J. 2012 Feb 15;11:47. doi: 10.1186/1475-2875-11-47 (PMC3306729; doi:10.1186/1475-2875-11-47)
Supplement: Additional file 1 — Table S1. Cytokine association tests according to TLR2 genotype in Uganda children with cerebral malaria or uncomplicated malaria. [file 1475-2875-11-47-S1.DOC]

Supplementary Table 1: Cytokine association tests according to *TLR2* genotype in Uganda children with cerebral malaria or uncomplicated malaria

|  |  |  |  | p value | |
| --- | --- | --- | --- | --- | --- |
| cytokine | polymorphism | Model | genotypes | CM | UM |
| logIFN-γ | Δ22 | General | WW vs WD vs DD | 0.523 | 0.942 |
|  | Δ22 | Dominant | WW vs WD/DD | 0.789 | 0.748 |
|  | Δ22 | Recessive | DD vsWD/WW | 0.437 | 0.994 |
|  | Δ22 | Additive | WW vs WD | 0.433 | 0.740 |
|  | Δ22 | Additive | WW vs DD | 0.549 | 0.941 |
|  | GTn | General | SM vs MM vs ML | 0.057 | 0.720 |
|  | GTn |  | MM vs SM/ML | 0.502 | 0.788 |
|  | GTn |  | SM vs ML | 0.119 | 0.473 |
|  | GTn |  | SM vs MM/ML | 0.530 | 0.402 |
|  | GTn |  | ML vs MM/SM | 0.137 | 0.692 |
| LogTNF | Δ22 | General | WW vs WD vs DD | 0.450 | 0.541 |
|  | Δ22 | dominant | WW vs WD/DD | 0.382 | 0.313 |
|  | Δ22 | Recessive | DD vsWD/WW | 0.653 | 0.338 |
|  | Δ22 | Additive | WW vs WD | 0.175 | 0.344 |
|  | Δ22 | Additive | WW vs DD | 0.862 | 0.202 |
|  | GTn | General | SM vs MM vs ML | 0.404 | 0.071 |
|  | GTn |  | MM vs SM/ML | 0.988 | 0.416 |
|  | GTn |  | SM vs ML | 0.333 | 0.076 |
|  | GTn |  | SM vs MM/ML | 0.393 | 0.171 |
|  | GTn |  | ML vs MM/SM | 0.414 | **0.007** |
| logIL1β | Δ22 | General | WW vs WD vs DD | 0.708 | 0.174 |
|  | Δ22 | dominant | WW vs WD/DD | 0.888 | **0.029** |
|  | Δ22 | Recessive | DD vsWD/WW | 0.359 | **0.030** |
|  | Δ22 | Additive | WW vs WD | 0.661 | **0.029** |
|  | GT_n_ | General | SM vs MM vs ML | 0.344 | 0.483 |
|  | GT_n_ |  | MM vs SM/ML | 0.961 | 0.055 |
|  | GT_n_ |  | SM vs MM/ML | 0.478 | 0.055 |
|  | GT_n_ |  | ML vs MM/SM | **0.000** | 0.055 |
| logIL6 | Δ22 | General | WW vs WD vs DD | 0.798 | 0.239 |
|  | Δ22 | dominant | WW vs WD/DD | 0.606 | 0.669 |
|  | Δ22 | Recessive | DD vsWD/WW | 0.547 | **0.041** |
|  | Δ22 | Additive | WW vs WD | 0.774 | 0.455 |
|  | Δ22 | Additive | WW vs DD | 0.513 | 0.063 |
|  | GT_n_ | General | SM vs MM vs ML | 0.530 | 0.384 |
|  | GT_n_ |  | MM vs SM/ML | 0.383 | 0.925 |
|  | GT_n_ |  | SM vs ML | 0.542 | 0.117 |
|  | GT_n_ |  | SM vs MM/ML | 0.728 | 0.394 |
|  | GT_n_ |  | ML vs MM/SM | 0.385 | 0.120 |
| logIL10 | Δ22 | General | WW vs WD vs DD | 0.559 | 0.853 |
|  | Δ22 | dominant | WW vs WD/DD | 0.528 | 0.813 |
|  | Δ22 | Recessive | DD vsWD/WW | 0.463 | 0.735 |
|  | Δ22 | Additive | WW vs WD | 0.807 | 0.891 |
|  | Δ22 | Additive | WW vs DD | 0.443 | 0.718 |
|  | GT_n_ | General | SM vs MM vs ML | 0.577 | 0.461 |
|  | GT_n_ |  | MM vs SM/ML | 0.390 | 0.194 |
|  | GT_n_ |  | SM vs ML | 0.633 | 0.920 |
|  | GT_n_ |  | SM vs MM/ML | 0.405 | 0.125 |
|  | GT_n_ |  | ML vs MM/SM | 0.633 | 0.920 |
